# Cost-Effectiveness and Distributional Impact of Opportunistic Screening for People at High-Risk of Cardiovascular Disease in Sri Lanka: A Modelling Study

**DOI:** 10.5334/gh.1174

**Published:** 2022-12-22

**Authors:** Nilmini Wijemunige, Ravindra P Rannan-Eliya, Jürgen Maurer, Owen O’Donnell

**Affiliations:** 1 Institute for Health Policy, 72 Park Street, Colombo 2, Sri Lanka; 2 Department of Economics, University of Lausanne, Internef, Dorigny, 1015, Lausanne, Switzerland; 3 Erasmus School of Economics and Erasmus School of Health Policy & Management, Erasmus University Rotterdam, Postbus 1738, 3000 DR Rotterdam, The Netherlands

**Keywords:** Cardiovascular diseases, hypertension, diabetes, screening, equity, Sri Lanka

## Abstract

**Background:**

While hypertension, diabetes, hypercholesterolemia and high-risk of cardiovascular disease can be easily diagnosed and treated with cost-effective medicines, a large proportion of people remain undiagnosed. We assessed the potential effectiveness, cost, and distributional impact of opportunistically screening for these chronic conditions at outpatient patient departments in Sri Lanka.

**Methods:**

We used nationally representative data on biomarkers and healthcare utilization in 2019 to model the screening of people aged 40+ without preexisting CVD and without a reported diagnosis of hypertension, diabetes, or hypercholesterolemia. We modelled an intensive one month program that would screen a proportion of those making an outpatient visit to a public or private clinic and follow-up a proportion of those screened to confirm diagnoses. We also modelled a less intensive one year program. The main outcomes were the new diagnoses of any of the chronic conditions. Program costs were calculated and the socioeconomic distributions of individuals screened, new cases diagnosed, and treatments delivered were estimated. Sensitivity analyses varied the probability of screening and follow-up.

**Results:**

Using data on 2,380 survey participants who met the inclusion criteria, we estimated that the one month program would diagnose 8.2% (95% CI: 6.8, 9.6) of those with a chronic condition who would remain undiagnosed without the program. The one year program would diagnose 26.9% (95% CI: 26.5, 27.4) of the otherwise undiagnosed and would have a cost per person newly diagnosed of USD 6.82 (95% CI: 6.61, 7.03) in the public sector and USD 16.92 (95% CI: 16.37, 17.47) in the private sector. New diagnoses would be evenly distributed over the socioeconomic distribution, with public (private) clinics diagnosing a higher proportion of poorer (richer) individuals. Both programs would reduce underdiagnosis among males relative to females.

**Conclusions:**

Opportunistic screening for cardiovascular diseases at outpatient clinics in Sri Lanka could be cost-effective and equitable.

## Introduction

In most low- and middle-income countries (LMICs), the burden of cardiovascular diseases is increasing [1], reaching 16% of all disability-adjusted life years (DALYs) in 2019 [2]. In Sri Lanka, this burden is even higher at 29% of DALYs, while ischemic heart disease, stroke, and diabetes mellitus accounted for 38% of mortality in 2019 [2]. In high-income countries, these conditions caused 21% of DALYs and 27% of mortality [2]. Screening for these diseases and their risk factors can hasten diagnosis, treatment, and control, and substantially reduce premature, avertable mortality [3]. Diagnosis and management of hypertension, diabetes, and high-risk of CVD are considered ‘best-buys’ [4], and is an important component of the Package of Essential Noncommunicable Disease Interventions for Primary Health Care in Low Resource Settings (PEN) [5]. In programs screening for high-risk of CVD, risk factors, including hypertension, diabetes, and hypercholesterolemia, are detected and treated, which by extension, treats and reduces CVD risk [5].

In 2016, Sri Lanka’s Ministry of Health (MOH) set targets to reduce the prevalence of hypertension by 25% and to reduce mortality due to diabetes and CVD by 20% by 2025 [6]. It has set up 1,000 dedicated clinics–Healthy Lifestyle Centres–capable of screening nearly one million people a year for risk factors which lead to CVD [7,8,9]. The screening assesses hypertension status, diabetes status, and CVD risk in people aged 35 and over without pre-existing CVD [9]. While numbers screened have been increasing, only 605,000 people (7% of the target population) were screened in 2019, and only 28% of those screened were male [9].

Despite the potential for opportunistic screening for cardiovascular disease risk factors at routine medical consultations to deliver cost-effective interventions that help reorient primary care toward management of chronic diseases, it is not common in LMICs [3,10]. In Sri Lanka, where there is frequent use of outpatient (OP) care–around seven to eight visits per person per year [11]–opportunistic screening can potentially reach a much larger proportion of the target population.

This study aimed to assess the effectiveness, cost, and distributional impact of an opportunistic screening program for high-risk of CVD, along with hypertension, diabetes and hypercholesterolemia, implemented through OP visits of Sri Lankans aged 40 years and older without pre-existing CVD, and without a previous diagnosis of hypertension, diabetes or hypercholesterolemia.

## Materials and methods

### Survey design and measurements

We modelled the screening program using data on prevalence of CVD-related chronic conditions, their diagnoses, and healthcare utilization from the 2018/2019 Sri Lanka Health and Ageing Study (SLHAS). This was a nationally representative, stratified, multi-stage cluster random sample of adults aged 18 years and older. Participants attended a field clinic close to their residence where a questionnaire was completed and biomarkers were measured [12]. A medical history of hypertension, diabetes, hypercholesterolemia, and CVD was taken, along with self-reported use of healthcare and medication. Medical records, if brought to the field clinic, were checked for medications prescribed and diagnoses of hypertension, diabetes, hypercholesterolemia or CVD.

Each participant had their blood pressure (BP) measured, fasting blood glucose, and lipid profiles (see Supplement 1). Most participants were asked how many OP visits they made to various types of healthcare facilities in a 28-day recall period. Others were randomly assigned to report OP visits in one of two other recall periods.

### Classification

For the modelling exercise, we used the data on biomarkers, reported diagnoses and medication, medical records and criteria given in Table 1 to determine whether each participant had and was already diagnosed with any of three chronic conditions: i) hypertension, ii) diabetes, and iii) hypercholesterolemia or high-risk of CVD. A participant was ‘undiagnosed’ if they fulfilled any criterion for ‘has condition’ but did not satisfy any criterion for ‘already diagnosed’. We grouped hypercholesterolemia and high-risk of CVD together as one condition since either is sufficient to prescribe statins according to respective guidelines [12]. Participants with pre-existing CVD, which we defined as participants who reported or had medical records consistent with having been diagnosed with angina, coronary artery disease or myocardial infarction, were not eligible for screening.

**Table 1:** Criteria used to define disease state and diagnosis status of the chronic conditions of SLHAS participants for inclusion in modelling.


CRITERIA BY CONDITION	HAS CONDITION	ALREADY DIAGNOSED

Hypertension		

**a)** reported having been diagnosed with hypertension or their medical records showed this	✓	✓

**b)** reported taking antihypertensives in the past 14 days	✓	✓

**c)** had a systolic blood pressure of 140 mmHg or more, or a diastolic blood pressure of 90 mmHg or more	✓	

Diabetes		

**a)** reported having been diagnosed with diabetes or their medical records showed this	✓	✓

**b)** reported taking oral hypoglycemics or insulin in the past 14 days	✓	✓

**c)** had a fasting blood glucose ≥ 126 mg/dL, a random glucose ≥ 200 mg/dL, or an oral glucose tolerance test result ≥ 200 mg/dL	✓	

Hypercholesterolemia or high CVD risk		

**a)** reported having been diagnosed with hypercholesterolemia or their medical records showed this	✓	✓

**b)** reported taking a statin in the past 14 days	✓	✓

**c)** had a total cholesterol of 300 mg/dL or more	✓	

**d)** had a 10-year CVD risk based on the 2019 WHO risk charts (26) of 20% or more	✓	


*Notes*: Only one applicable criteria under each condition needs to be fulfilled for a participant to be categorized as ‘has condition’ and ‘already diagnosed’. Criteria c) and d) are referred to as biomarker data in Calculations.

We identified OP visits to public and private specialist clinics, public and private general clinics, and public Medical Officer of Health clinics as those at which opportunistic screening could potentially be initiated.

### Intervention

**Figure 1:**
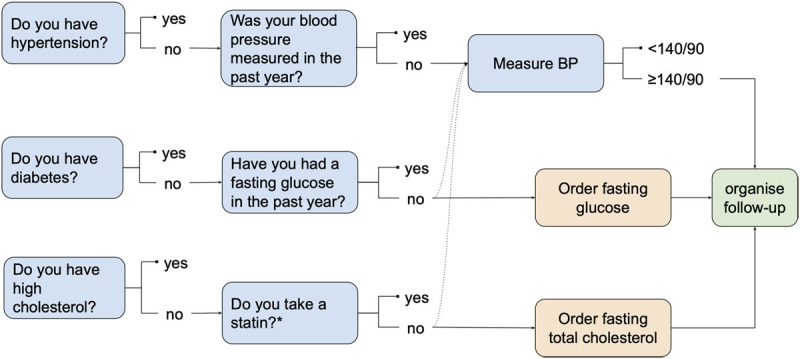
Processes of the screening program for people without preexisting CVD.

We modelled a screening process (Figure 1, Supplement 1) that incorporates steps specified in the Sri Lankan Ministry of Health guidelines for screening [12]. It begins with simple questions, similar to those asked in the SLHAS survey, for people without preexisting CVD, to ascertain whether a patient has a history of hypertension, diabetes or hypercholesterolemia. Negative answers trigger a further set of questions or BP measurement and ordering of a fasting glucose or cholesterol test, as appropriate. In most cases, a second follow-up appointment is arranged, either at the same clinic for a regular patient or at a nearby Healthy Lifestyle Centre, to repeat measurement of BP, review fasting glucose and cholesterol results, calculate CVD risk based on these measurements, diagnose hypertension or diabetes, determine whether a statin is required based on cholesterol and/or predicted CVD risk and explain the management plan to the patient where needed.

We modelled a national program in which initial screenings would take place within a 28-day period which could be pitched as a CVD-riskscreening month. We assumed that screening would occur in 60% of OP visits to public facilities and 55% of OP visits to private clinics. These rates were based on estimates that process quality of care indicators, such as measuring BP in a known hypertensive patient or measuring blood glucose in a diabetic patient, were met in 70% and 65% of relevant OP consultations in the public and private sectors, respectively [13]. This parameter was changed in sensitivity analyses.

In addition to a 28-day program, we modelled a program that would run less intensively for one year. In this program, we assumed screening would occur in 30% and 28% of OP visits to public and private facilities (that is, half the probability of the 28-day model), respectively. This program would place less demands on the day-to-day operation of clinics, but it would run over a longer period.

We assumed that 60% of those who would be screened in both the 28-day and one-year programs would attend a follow-up visit irrespective of health status, sociodemographic characteristics, and type of facility initially visited, with this parameter varied in sensitivity analyses from 40% to 80%. A small intervention trial in the US, found that over 60% of participants who were screened and had been told they were at elevated risk for CVD had visited a doctor within three months [14].

### Outcomes

The main outcomes were the number of people newly diagnosed with any of the three chronic conditions, this number as a proportion of those who would remain undiagnosed without the program, and the cost per person newly diagnosed. In secondary analysis, we estimated the number and proportion newly diagnosed for each of the three conditions separately.

### Calculations

We used binomial probabilities to calculate the probability that a participant would get the initial screen based on the number of OP visits reported over a 28-day period (Supplement 1). For the one-year program, we did not have data on OP visits over a one-year period. Separately for public and private OP visits and by age, sex, and socioeconomic group, we estimated the proportion of participants that would have an OP visit in a year by extrapolating from a model of how the probability of having an OP visit varied for a seven-day, a 14-day and a 28-day recall period (see Supplement 1).

The number of patients screened was calculated by multiplying each participant’s sample weight () by their probability of being screened () and summing over all participants. The weights scale the sample and make it representative of the population of Sri Lanka aged 40 years and over. We then calculated the number of patients followed-up by multiplying the number screened by the probability of follow-up (0.6). For each of the chronic conditions, the number of people that would be newly diagnosed by the screening program was the number followed-up who were identified to have that condition using the biomarker data but who were previously undiagnosed (Table 1). We also calculated the proportions of people with a chronic condition according to biomarker data but were undiagnosed and would be newly diagnosed by the screening program. For each chronic condition, we calculated how many were already diagnosed, how many would be newly diagnosed in the 28-day program, how many would be additionally diagnosed in the one-year program, and who would not be diagnosed in either program.

Following the same general procedure, we calculated the number of newly diagnosed cases identified through opportunistic screening at OP visits to public and private facilities separately. The weight of a participant who visited both sectors was distributed proportionately based on the number of visits to each sector and the respective probability of screening. Costs were calculated separately for the public and private sectors. Costs for the public sector were from a government budgetary perspective, and covered consumable, reagent and labour costs for laboratory testing and the cost for a follow-up visit. Costs for the private sector were from a patient perspective, covering prices of laboratory tests and the cost for a follow-up visit, although private sector doctors often do not charge when followingup reports ordered at a previous appointment. Costs were based on prices quoted in the public and private sectors in 2021, which were converted to December 2021 US dollars (US$1 = LKR 201.40) [15].

We used concentration curves and concentration indices [16] to assess socioeconomic inequality in the distributions of undiagnosed cases before and after the opportunistic screening intervention, and to measure inequality in the distribution of newly diagnosed cases. We proxied socioeconomic status by a wealth index equal to the first principal component from analysis of a battery of household durable assets, housing quality, water and sanitation facilities, and other assets (Supplementary Table 3). A concentration curve traced the cumulative proportion of undiagnosed cases, for example, against the cumulative proportion of the sample ranked from the poorest according to the wealth index to the richest. A concentration curve above (below) the 45-degree line of equality indicates a disproportionate concentration of cases among the poor (rich). We measured inequality using a concentration index appropriate for a binary outcome [17]. A negative (positive) concentration index indicates inequality in the direction of the poor having more (less) of the outcome. We used a two-sample z-test to test a null hypothesis of equal proportions of newly diagnosed (or undiagnosed) between groups defined by the poorest and richest quintiles of the wealth index distribution.

### Sensitivity analysis

For both the 28-day and one-year programs, we evaluated the impact of varying the assumed probability of follow-up from 60% to 80% and 40%. We also modelled a one-year program that would have the same screening probabilities and so be as intensive as the 28-day program.

## Results

**Figure 2:**
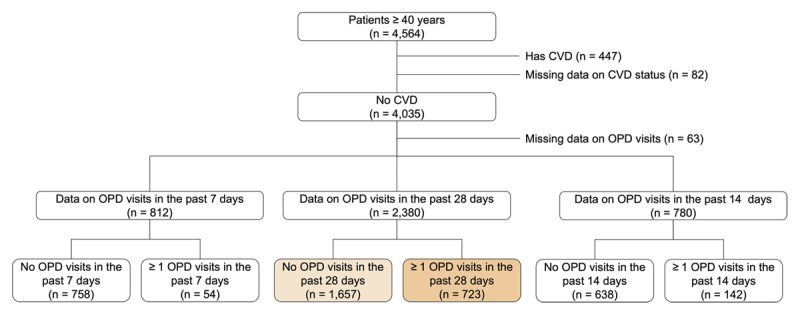
Participant flow.

Out of 6,668 participants aged 18+, 4,564 were aged 40+, and 4,035 of those in this age group had no history of CVD (Figure 2). After the loss of 63 participants with no data on OP visits, there were 3,972 participants in the analysis sample. Of these, 2,380 had data on OP visits in the past 28 days and were used to model the 28-day program. In this sub-sample of 2,380 people, 730 (31%) had at least one chronic condition that was undiagnosed (Table 2). Among those with an undiagnosed chronic condition, around 63% (458/730) had undiagnosed hypertension, 31% (228/730) had diabetes, and 33% (241/730) had hypercholesterolemia or high CVD risk. Of these 730 participants, 176 (24.1%) had more than one undiagnosed condition.

**Table 2:** Sample participants ≥40 years with an undiagnosed chronic condition, and percent with outpatient (OP) visits by sector.


UNDIAGNOSED CONDITION	NUMBER	PERCENT WITH ANY OP VISIT (%)		

		PUBLIC	PRIVATE	ANY

28-day period				

Hypertension	458	13.4	12.5	25.5

Diabetes	228	16.6	5.3	21.7

Hypercholesterolemia or high CVD risk	241	16.1	8.7	24.8

Any	730	15.0	10.7	25.4

1-year period				

Hypertension	749	40.2	30.5	58.5

Diabetes	368	40.1	30.6	58.5

Hypercholesterolemia or high CVD risk	398	40.6	30.2	58.6

Any	1,220	40.3	30.5	58.5


*Notes*: Percentages calculated from weighted sample.

Over 25% of those who had any undiagnosed chronic condition had an OP visit in the past 28 days (Table 2). Among participants with an undiagnosed chronic condition, higher percentages had visited public sector facilities.

Out of the 3,972 participants used to model the 1-year program—that is, people with no CVD and had data on whether they had a OP visit in the past seven, 14 or 28 days—31% (1,220/3,972) had an undiagnosed chronic condition. From the model, over half (59%) of those with an undiagnosed chronic condition would have at least one OP visit in a year.

Scaled to the Sri Lankan population, we estimated that there would be 2.32 million people (95% CI: 2.317, 2.322) without a previous diagnosis of CVD with an OP visit within a 28-day period that would be eligible for opportunistic screening (Table 3). Using the assumed probabilities of undergoing opportunistic screening when attending public and private sector clinics, we estimated that 1.4 million people (95% CI: 1.29, 1.53) would be assessed in a 28-day screening program. Assuming that 60% of those assessed and requested to return for a follow-up visit would make that visit, 666,000 (95% CI: 615, 718) would complete the screening process, and 192,000 (95% CI: 167, 217) would be newly diagnosed with one or more of the chronic conditions. Of those assessed, 13.6% (191,959/1,411,970) (95 CI: 11.0, 16.2) would be newly diagnosed with at least one chronic condition.

**Table 3:** Population estimates of people aged ≥40 years without pre-existing CVD, who are assessed, followed-up and newly diagnosed in 28-day and 1-year opportunistic screening programs.


	ELIGIBLE FOR SCREENING WITH ≥1 OP VISIT^†^ NO. (‘000S) (95% CI)	ASSESSED NO. (‘000S) (95% CI)	FOLLOWED-UP NO. (‘000S) (95% CI)	NEWLY DIAGNOSED		COST PER PERSON SCREENED (USD) (95% CI)	COST PER PERSON DIAGNOSED (USD) (95% CI)	TOTAL COST (USD ‘000) (95% CI)

				NO. (‘000S)(95% CI)	AS % OF ASSESSED^‡^ NO. (‘000S) (95% CI)			

28-day program								

Public	1,400 (1,398, 1,402)	857 (776, 939)	396 (350, 441)	123 (98, 148)	14.3 (11.0, 17.7)	1.01 (0.90, 1.13)	7.05 (6.24, 7.85)	867 (768, 966)

Private	976 (974, 978)	555 (483, 627)	271 (230, 311)	69 (49, 89)	12.4 (8.6, 16.2)	2.64 (2.32, 2.97)	21.27 (18.67, 23.88)	1,467 (1,288, 1,647)

All	2,320 (2,317, 2,322)	1,412 (1,292, 1,532)	666 (615, 718)	192 (167, 217)	13.6 (11.0, 16.2)	1.65 (1.52, 1.79)	12.16 (11.16, 13.16)	2,334 (2,141, 2,527)

1-year program								

Public	3,084 (3,007, 3,162)	2,191 (2,13, 2,248)	1,147 (1,112, 1,182)	375 (348, 401)	17.1 (15.9, 18.3)	1.17 (1.13, 1.20)	6.82 (6.61, 7.03)	2,555 (2,476, 2,634)

Private	2,336 (2,276, 2,396)	1,492 (1,450, 1,535)	770 (745, 796)	253 (235, 271)	17.0 (15.7, 18.2)	2.87 (2.78, 2.96)	16.92 (16.37, 17.47)	4,281 (4,143, 4,419)

All	4,483 (4,371, 4,596)	3,683 (3,587, 3,778)	1,918 (1,859, 1,976)	628 (584, 671)	17.0 (15.8, 18.2)	1.86 (1.80, 1.91)	10.89 (10.56, 11.23)	6,836 (6,625, 7,047)


*Notes*: ^†^ People aged 40 years and over without pre-existing CVD are eligible for screening, regardless of chronic condition status. ^‡^ Percentage of all people with an undiagnosed chronic condition, regardless of whether they reported an OP visit.

With a less intensive one-year program that would screen lower proportions of those with OP visits, we estimated that about 4.5 million (95% CI: 4.37, 4.60) patients would be eligible for screening, and 3.7 million (95% CI: 3.59, 3.78) of them would be assessed, with 17.0% (95% CI: 15.8, 18.2) of those assessed (627,531/3,682,674) being newly diagnosed with at least one chronic condition (Table 3).

**Figure 3:**
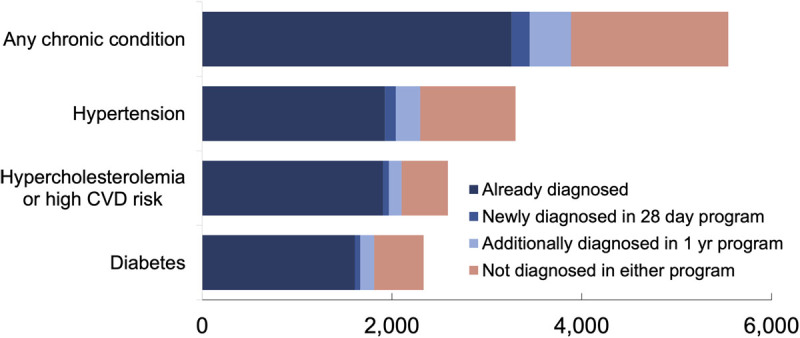
Population estimates of people with chronic conditions that are diagnosed before screening, diagnosed by screening and undiagnosed after screening in 28-day program and one-year program (’000s).

The 28-day program would identify 8.0% (95% CI: 6.1, 9.8) of undiagnosed hypertensives, 7.4% (95% CI: 5.2, 9.6) of undiagnosed diabetics and 8.7% (95% CI: 6.2, 11.3) of those with undiagnosed hypercholesterolemia or high CVD risk, in the population aged 40 years or more (Figure 3, Supplementary Table 4). Overall, 8.2% (191,959/2,331,756) (95% CI: 6.8, 9.6) of people with any undiagnosed chronic condition would be diagnosed. The one-year program was estimated to detect 26.9% (627,531/2,331,756) (95% CI: 26.5, 27.4) of people with any undiagnosed chronic condition. Males would comprise 42% (279,634/666,493) (95% CI: 37, 47) of those screened and followed-up (Supplementary Table 5).

With the 28-day program, the cost to the government per person screened in the public sector was estimated to be US$1.01 (95% CI: 0.90, 1.13), and the cost per person diagnosed was US$7.05 (95% CI: 6.24, 7.85) (Table 3). The costs in the one-year program are similar, where the public cost per person screened is US$1.17 (95% CI: 1.13, 1.20) and cost per diagnosis is US$6.82 (95% CI: 6.61, 7.03). The estimated total cost to the government was US$867,000 with the 28-day program and US$2,555,000 with the one-year program (Table 3), which is 0.07% and 0.21% of total public health expenditure in 2019, respectively [18]. The out-of-pocket costs for patients in the private sector was estimated to be US$2.64 (95% CI: 2.32, 2.97) per person screened in the 28-day program and US$2.87 (95 CI: 2.78, 2.96) in the one-year program, with the total spent being the equivalent of 0.12% and 0.35% of total private health expenditure, respectively [18] (Supplementary Table 6).

**Figure 4:**
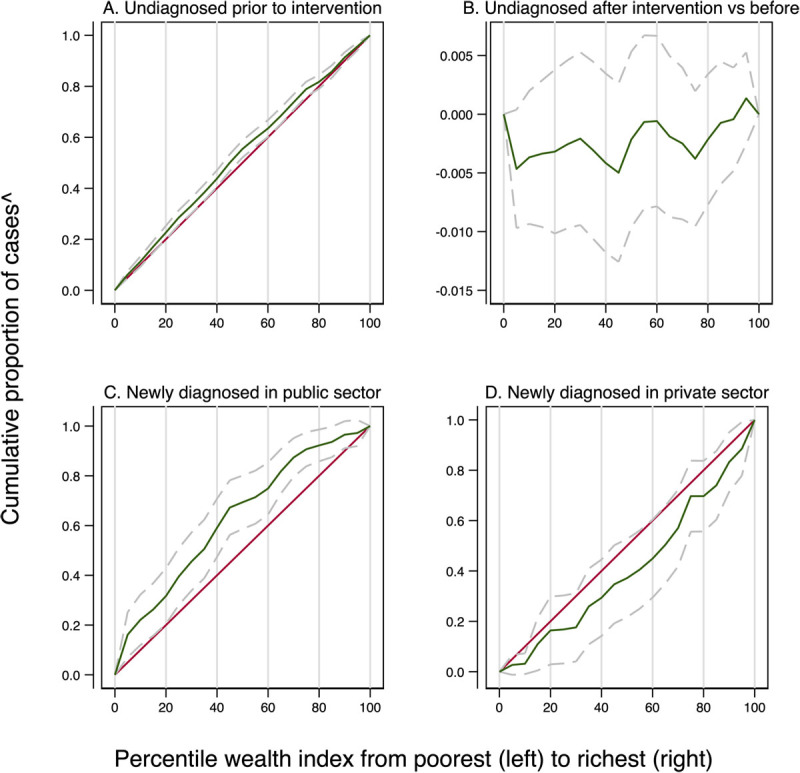
Concentration curves for undiagnosed cases before and after screening and for newly diagnosed cases. *Notes*: Concentration curves drawn for people aged 40 years and over without preexisting CVD, with at least one chronic condition. ^ y-axis for Figure B is the difference of the proportion undiagnosed after intervention and proportion undiagnosed before intervention. Grey dashed lines show the 95% confidence intervals of the concentration curves.

Table 4 shows the distributions of people eligible for screening, undiagnosed before and after screening, and newly diagnosed by screening by socioeconomic quintile, with estimated numbers shown in Supplementary Table 7. Of the people eligible for screening, 59% (95% CI: 53, 64) of the poorest quintile and 70% (95% CI: 65, 76) of the richest quintile had at least one chronic condition. Point estimates indicate that the percentage with an undiagnosed chronic condition was higher in the poorest quintile than in the richest quintile, although this difference is not significant (32.5% vs 29.5%, p = 0.4). The negative concentration index (C) (-0.11; 95% CI: -0.16, -0.05; p < 0.001) confirms that poorer individuals with a chronic condition were more likely to be undiagnosed before screening with the 28-day program, which is also demonstrated by a concentration curve that lies above the 45-degree line in Figure 4A. We estimated that after implementation of this program the distribution of undiagnosed chronic conditions amongst those with a chronic condition would become very slightly less concentrated on poorer individuals, which is indicated by a concentration index that is smaller in magnitude (C = -0.09; 95% CI: -0.14, -0.04; p = 0.001) and less undiagnosed people in the poorer quintiles following the intervention (Figure 4B). However, neither the concentration indices nor the concentration curves are significantly different. We estimated that new diagnoses of any chronic condition identified through screening at public clinics would be slightly skewed toward the poor (C = -0.03; 95% CI: -0.04, -0.02; p < 0.001), confirmed by the concentration curve above the 45-degree line (in Figure 4C) while private clinics would make slightly more new diagnoses of richer individuals (C = 0.01; 95% CI 0.002, 0.018; p = 0.013, confirmed by the concentration curve below the 45-degree line (in Figure 4D). Overall, considering both public and private sectors, new diagnoses would be slightly more concentrated among poorer individuals (C = -0.02; 95% CI: -0.03, -0.004; p = 0.01).

**Table 4:** Distributions of screening eligible, undiagnosed and newly diagnosed individuals by socioeconomic status with 28-day screening program.


SES QUINTILE	ELIGIBLE FOR SCREENING^†^ NO. (‘000S)(95% CI)	ELIGIBLE FOR SCREENING WITH ≥1 CHRONIC CONDITION % (95% CI)	UNDIAGNOSED		NEWLY DIAGNOSED		
	
			BEFORE SCREENING% (95% CI)	AFTER SCREENING% (95% CI)	PUBLIC% (95% CI)	PRIVATE% (95% CI)	BOTH% (95% CI)

1 (poorest)	1,536 (1,424, 1,647)	58.7 (53.2, 64.2)	32.5 (27.2, 37.8)	29.2 (24.5, 34.0)	2.5 (1.4, 3.7)	0.7 (0.0, 1.4)	3.3 (2.0, 4.5)

2	1,533 (1,423, 1,642)	58.8 (53.1, 64.4)	29.1 (23.8, 34.3)	26.9 (21.9, 32.0)	1.6 (0.9, 2.3)	0.5 (0.0, 1.0)	2.1 (1.3, 3.0)

3	1,528 (1,428, 1,628)	62.9 (57.4, 68.4)	31.3 (26.1, 36.5)	29.0 (24.0, 33.9)	1.6 (0.7, 2.6)	0.7 (0.2, 1.2)	2.3 (1.3, 3.4)

4	1,531 (1,427, 1,636)	65.4 (59.9, 70.9)	29.9 (24.6, 35.2)	27.1 (22.2, 32.0)	1.6 (0.8, 2.5)	1.2 (0.5, 1.9)	2.8 (1.8, 3.9)

5 (richest)	1,532 (1,417, 1,647)	70.1 (64.8, 75.5)	29.5 (24.1, 34.8)	27.5 (22.4, 32.5)	0.6 (0.1, 1.2)	1.4 (0.6, 2.1)	2.0 (1.1, 2.9)

Total	7,660 (7,418, 7,902)	63.2 (60.7, 65.6)	30.4 (28.1, 32.8)	27.9 (25.7, 30.1)	1.6 (1.2, 2.0)	0.9 (0.6, 1.2)	2.5 (2.0, 3.0)

Concentration index (95% CI)							

	Eligible with ≥ 1 chronic condition^‡^	Not applicable	–0.106 (–0.164, –0.049)p < 0.001	–0.089 (–0.143, –0.035)p = 0.001	–0.027 (-0.038, –0.016)p < 0.001	0.010 (0.002, 0.018)p = 0.013	–0.017 (–0.030, –0.004)p = 0.010

	Eligible*	0.100 (0.055–0.145)p < 0.001	–0.019 (–0.062, 0.023)p = 0.375	-0.012 (–0.052, 0.027)p = 0.541	–0.014 (–0.021, –0.008)p < 0.001	0.008 (0.003, 0.013)p = 0.003	–0.007 (–0.015, 0.002)p = 0.111


*Notes*: ^†^ People aged 40 years and over without pre-existing CVD. ^‡^ Concentration index calculated on all people aged 40 years and over without preexisting CVD, and with at least one chronic condition. *Concentration index calculated on all people aged 40 years and over without preexisting CVD. SES = socioeconomic status.

Estimates for the sensitivity analyses are shown in Supplementary Table 5 and 6. The cost per person diagnosed ranged from US$6.44 to US$7.73 in the public sector and US$15.03 to US$25.75 in the private sector. A more intensive one-year program, where probability of assessment was doubled to 60% in the public sector and 55% in the private sector, marginally increased the percentage of newly diagnosed cases from 27% to 32%, with the total cost to the government increasing by a similar amount (16%). The program with the most impact is a one-year program with high probabilities of screening per encounter (60% in the public sector and 55% in the private sector), as well as a high follow-up rate of 80%. Such a program would newly diagnose 42% of undiagnosed cases; the costs per person diagnosed is similar to the one-year base case, and the total cost for the public health sector would be 0.31% of the 2019 annual expenditure on health by the government. The cost per person diagnosed in the government sector reduced by 5% if the follow-up rate increased from 60% to 80%, and increased by about 10% if the follow-up rate reduced from 60% to 40% in both the 28-day and one-year scenarios. For example, in the one-year scenario where the probability of being screened is 30% in the public sector and 28% in the private sector, if the probability of follow-up was lowered from 60% to 40%, the proportion of undiagnosed people that would be newly diagnosed over the course of the year reduces from 27% to 18%, with a 10% increase in the cost per diagnosis from US$6.82 to US$7.53.

## Discussion

Opportunistic screening at healthcare encounters has been proposed to increase detection of chronic conditions in LMICs [3,19]. However, there was a lack of evidence on the effectiveness, cost, and distributional impact of such.

Our study finds that opportunistic screening could moderately increase the detection of people with undiagnosed chronic conditions. With a pragmatic 28-day program in which 60% of OP patients at public clinics and 55% of OP patients at private clinics would be assessed, and only 60% followed-up, we estimated that 8% of people with an undiagnosed chronic condition would be detected. With a one-year program in which the probabilities of assessment on a single encounter were lowered to 30% and 20% for public and private clinics, respectively, 27% of those with an undiagnosed chronic condition would be detected. Furthermore, the distribution of new diagnoses was broadly distributionally neutral: overall, the distribution of people with an undiagnosed chronic condition would become slightly less skewed towards the poor. The detection of new diagnoses would be slightly pro-poor at public clinics and slightly pro-rich at private clinics. Whilst the government could introduce screening that is pro-poor in the public sector, it is likely that there will be spill-on effects in the private sector since most physicians in the private sector are government doctors engaged in dual practice [13].

A major advantage of opportunistic screening over community-based screening is that it makes use of doctors and facilities that are already available. Although such a screening program would require additional resources at several stages, most requirements are likely to be manageable. First, the initial assessment, which involves asking patients a simple set of questions, taking physical measurements, and arranging laboratory tests and follow-up, would require only a slight lengthening of the duration of existing consultations, and could potentially be carried out by several types of healthcare staff, particularly in the public sector [20]. Furthermore, screening would take place in only 3.7% of all public OP visits (2.2 million out of 58.7 million visits in 2019 [9]) in the one-year program. The private sector generally has longer consultation times, which may be compatible with a quick assessment [13]. Whilst it is difficult to ascertain the burden on laboratory testing, the envisaged cost of laboratory supplies for the one-year program in the government sector is 1% of the total government laboratory supplies expenditure reported in 2019 [9], suggesting it would be feasible to absorb laboratory testing of publicly assessed participants in the public sector, with labour for testing costed as well. The number of visits required for follow-up in the public sector would represent, at most, a 2% increment to the total OP visits in 2019 [9], assuming that they cannot be absorbed into existing follow-up visits and the underutilized capacity of Healthy Lifestyle Centres of 200,000 patients a year. Though this may require extra planning prior to launching a large-scale screening program, given that there was a 2.4% annual increase in public OP visits between 2008 and 2019, it is possible the system would be able to absorb the additional follow-up visits needed [9,21]. Nevertheless, the cost of follow-up, including personnel, infrastructure and indirect costs, was included for both sectors.

Even in the intensive one-year screening program, the total cost to the government was estimated at USD 3,745,000, which is 0.31% of the total annual public health expenditure for 2019 [18]. A one-year program with a modest screening probability can diagnose more people, at less cost per diagnosis, than a high-intensity one month program.

We modelled a uniform follow-up rate by disease condition because the conditions considered are largely asymptomatic, and there is no disease-based reason to expect a differential in follow-up visit rates. However, follow-up rates may vary, particularly by gender and socioeconomic status. In the sensitivity analysis, higher follow-up rates would reduce the cost per person diagnosed and proportionately increase the percentage of new diagnoses. However, even a one-year program with a probability of screening of 30% and 28% per public and private sector encounter, and a low follow-up rate of 40%, would still newly diagnose 18% of undiagnosed people, with a marginal increase in cost per diagnosed person in the public sector compared to the base case. To increase the impact of the program, reduce cost per diagnosis, and ensure that any expansion of the health system is fully utilized, it is imperative the follow-up rates be increased as much as possible.

Whilst richer people were more likely to have a chronic condition, poorer people were more likely be undiagnosed. This is similar to other LMICs [22,23] with authors hypothesizing that higher diagnosis rates in the rich may be due to better access to screening [22]. Diagnoses from opportunistic screening at public clinics would slightly more concentrated on poorer people. However, poorer people would predominate among those who remained undiagnosed after opportunistic screening, with the extent of this inequality depending on how intensive the screening programs in the public and private sectors would be and how much follow-up would be achieved in each sector and socioeconomic group. The opportunistic screening program would also likely diagnose a greater proportion of males than is currently the case at Health Lifestyle Centres. The projected reductions in inequalities in diagnosis are consistent with a study which found opportunistic screening for hypertension in LMICs would reduce female-male and urban-rural differences in diagnosis of hypertension [10].

There are several limitations to this study. The estimates of doctor visits for the one-year program relied on modelling, although a subanalysis suggests that our model gives a conservative estimate of the number of people who had an OP visit in the past year (Supplement 1). As Sri Lanka undergoes an economic crisis with a depreciating currency and foreign exchange shortage resulting in medicine shortages, advocating for screening programs may be challenging in the short-term [24], given that up to 86% of those assessed would not be ultimately diagnosed with chronic conditions, and the need for initiating longterm treatment for those who reach treatment thresholds. However, implementing cost-effective screening and treatment programs for those with chronic conditions will help reduce the long-term impact and costs of undiagnosed chronic disease [19]. Lastly, this study does not assess treatment and control, which are imperative to reduce the burden of disease.

The current community-based screening program for chronic conditions is problematic with limited penetration and systematic difficulty in reaching men. A key strength of this study is that it demonstrates that in a country with relatively low health spending, and where each person on average visits a doctor seven to eight times a year (similar to the OECD average [25]), and 30% of people eligible for screening have at least one undiagnosed chronic condition, the use of opportunistic screening for the four chronic conditions could newly diagnose a sizeable number of people in an equitable way, for relatively low cost.

## Conclusion

The modelling exercise showed that it would be affordable, likely feasible and effective to screen opportunistically for people at high-risk for CVD. Furthermore, such a screening program would address a gender disparity diagnosis by increasing the diagnosis of males disproportionately, and it would slightly reduce socioeconomic inequality in diagnosis. It is important to assess whether the public health system would be able to absorb an estimated 2% increase in outpatient visits arising from the program.

## Data Accessibility Statement

The data used in this article are subject to an embargo until late-2022 in accordance with the overall Sri Lanka Health and Ageing Study Open Data policy. Once the embargo expires the data will be shared on reasonable request to the corresponding author.

## Additional File

The additional file for this article can be found as follows:

10.5334/gh.1174.s1 Supplemental Material. Supplement 1 to 3.

## References

[1] Roth GA, Mensah GA, Johnson CO, Addolorato G, Ammirati E, Baddour LM, et al. Global burden of cardiovascular diseases and risk factors, 1990–2019: Update from the GBD 2019 study. Journal of the American College of Cardiology. 2020; 76(25): 2982–3021. DOI: 10.1016/j.jacc.2020.11.010 33309175 PMC7755038

[2] Global Burden of Disease Study 2019 (GBD 019) Results [Internet]. Institute for Health Metrics and Evaluation (IHME). Available from: http://ghdx.healthdata.org/gbd-results-tool (accessed 29 April 2021).

[3] Bovet P, Chiolero A, Paccaud F, Banatvala N. Screening for cardiovascular disease risk and subsequent management in low and middle income countries: challenges and opportunities. Public Health Reviews. 2015; 36: 13. DOI: 10.1186/s40985-015-0013-0 29450041 PMC5804497

[4] World Health Organisation. Scaling up action against noncommunicable diseases: How much will it cost? Geneva: World Health Organisation; 2011.

[5] World Health Organisation. WHO package of essential noncommunicable (PEN) disease interventions for primary health care. Geneva: World Health Organisation; 2020.

[6] Ministry of Health. National multisectoral action plan for the prevention and control of noncommunicable diseases. Colombo: Ministry of Health; 2016.

[7] Mallawaarachchi DSV, Wickremasinghe SC, Somatunga LC, Siriwardena VT, Gunawardena NS. Healthy lifestyle centres: A service for screening noncommunicable diseases through primary health-care institutions in Sri Lanka. WHO South-East Asia Journal of Public Health. 2016; 5(2): 89–95. DOI: 10.4103/2224-3151.206258 28607234

[8] Ministry of Health SL. Annual health bulletin, 2018. Colombo: Ministry of Health; 2020.

[9] Ministry of Health SL. Annual health bulletin, 2019. Colombo: Ministry of Health; 2021.

[10] Maurer J, Ramos A. One-year routine opportunistic screening for hypertension in formal medical settings and potential improvements in hypertension awareness among older persons in developing countries: Evidence from the study on global ageing and adult health (SAGE). American Journal of Epidemiology. 2015; 181(3): 180–4. DOI: 10.1093/aje/kwu339 25550358 PMC4312429

[11] P4H Social Health Protection Network. National UHC dynamics card Sri Lanka 2020. Available from: https://p4h.world/en/national_uhc_dynamics_card_sri-lanka (accessed 28 June 2021).

[12] Ministry of Health. Cardiovascular risk management (total cardiovascular risk assessment approach). Guidelines for primary health care providers. Colombo: Ministry of Health; 2018.

[13] Rannan-Eliya RP, Wijemanne N, Liyanage IK, Jayanthan J, Dalpatadu S, Amarasinghe S, et al. The quality of outpatient primary care in public and private sectors in Sri Lanka—How well do patient perceptions match reality and what are the implications? Health Policy and Planning. 2015; 30 Suppl 1: i59–74. DOI: 10.1093/heapol/czu115 25355069

[14] Edelman DJ, Gao Q, Mosca L. Predictors and barriers to timely medical follow-up after cardiovascular disease risk factor screening according to race/ethnicity. Journal of the National Medical Assocation. 2008; 100(5): 534–9. DOI: 10.1016/S0027-9684(15)31299-2 PMC2704461 18507205

[15] Central Bank of Sri Lanka. Indicative US dollar SPOT exchange rate (LKR per 1 USD). Colombo; 2021.

[16] O’Donnell O, Van Doorslaer E, Wagstaff A, Lindelow M. Analyzing health equity using household survey data: A guide to techniques and their implementation. Washington, DC: The International Bank for Reconstruction and Development/The World Bank; 2008. DOI: 10.1596/978-0-8213-6933-3

[17] Erreygers G. Correcting the concentration index. Journal of Health Economics. 2009; 28(2): 504–15. DOI: 10.1016/j.jhealeco.2008.02.003 18367273

[18] Amarasinghe S, Dalpatadu K, Rannan-Eliya R. Sri Lanka health accounts: National health expenditure 1990–2019. Colombo: Institute for Health Policy; 2021.

[19] Beaglehole R, Epping-Jordan J, Patel V, Chopra M, Ebrahim S, Kidd M, et al. Improving the prevention and management of chronic disease in low-income and middle-income countries: A priority for primary health care. Lancet. 2008; 372(9642): 940–9. DOI: 10.1016/S0140-6736(08)61404-X 18790317

[20] Lim SS, Gaziano TA, Gakidou E, Reddy KS, Farzadfar F, Lozano R, et al. Prevention of cardiovascular disease in high-risk individuals in low-income and middle-income countries: Health effects and costs. Lancet. 2007; 370(9604): 2054–62. DOI: 10.1016/S0140-6736(07)61699-7 18063025

[21] Ministry of Health SL. Annual health bulletin, 2008. Colombo: Ministry of Health; 2009. DOI: 10.21315/mjms2019.26.5.9

[22] Lim OW, Yong CC. The risk factors for undiagnosed and known hypertension among Malaysians. Malaysian Journal of Medical Science. 2019; 26(5): 98–112. 10.21315/mjms2019.26.5.9 PMC6839659 31728122

[23] Mohanty SK, Upadhyay AK, Shekhar P, Kampfen F, O’Donnell O, Maurer J. Missed opportunities for hypertension screening: A cross-sectional study, India. Bull World Health Organ. 2022; 100(1): 30–9B. DOI: 10.2471/BLT.21.287007 35017755 PMC8722631

[24] Matthias AT, Jayasinghe S. Worsening economic crisis in Sri Lanka: Impacts on health. The Lancet Global Health. 2022. DOI: 10.1016/S2214-109X(22)00234-0 PMC9098208 35569487

[25] OECD/World Health Organization. Health at a glance: Asia/Pacific 2020: Measuring progress towards universal health coverage. Paris: OECD Publishing; 2020.

[26] The WHO CVD Risk Chart Working Group. World Health Organization cardiovascular disease risk charts: Revised models to estimate risk in 21 global regions. Lancet Global Health. 2019; 7(10): e1332–e45. 31488387 10.1016/S2214-109X(19)30318-3 PMC7025029

