# Supplementary material for: Cost-Effectiveness and Distributional Impact of Opportunistic Screening for People at High-Risk of Cardiovascular Disease in Sri Lanka: A Modelling Study
Source: Glob Heart. 2022 Dec 22;17(1):89. doi: 10.5334/gh.1174 (PMC9784145; doi:10.5334/gh.1174)
Supplement: Supplemental Material. — Supplement 1 to 3. [file gh-17-1-1174-s1.pdf]

Supplementary material

Cost-Effectiveness and Distributional Impact of Opportunistic Screening for People at High-Risk of Cardiovascular Disease in Sri Lanka: a Modelling Study

Nilmini Wijemunige, Ravindra Rannan-Eliya, Jürgen Maurer, Owen O'Donnell

Supplement 1

Additional information on survey design and measurements

Participants in the Sri Lankan Health and Ageing Study (SLHAS) were asked to fast for 10 to 12 hours, and venous samples were taken soon after clinic arrival. Those who did not report diabetes had an oral glucose tolerance test: after the fasting samples were taken, participants drank the equivalent of 75 grams of anhydrous glucose in solution, and a repeat venous sample was taken two hours later. Plasma and serum were extracted from the venous samples in the field and transported at -30 degrees Celsius to the study laboratory at the Medical Research Institute in Colombo, where they were analyzed, usually within two weeks of sample collection.

Additional information on intervention

We modeled a screening program for people aged 40 years or older who did not have a self-reported or recorded history of CVD. A person was defined as eligible for screening following Figure 1. In more detail, they were defined as eligible for screening if they fulfilled any one of:

a) no reported or recorded diagnosis of hypertension, not taking antihypertensives in the past 14 days, and no measurement of blood pressure by a health worker in the past year;

b) no reported or recorded diagnosis of diabetes, not taking oral hypoglycemics or insulin in the past 14 days, and no fasting glucose test (irrespective of result) conducted in the past year;

c) no reported or recorded diagnosis of hypercholesterolemia, and not taking a statin in the past 14 days.

These selection criteria are consistent with how the MOH guidelines for CVD screening in Sri Lanka identifies the target population. There is a slight difference in criteria for selecting participants in our model for screening, versus the criteria for identifying if a patient "has condition" for diabetes in Table 1. We did not include a recent OGTT (oral glucose tolerance test) as part of the modeling criterion for diabetes as SLHAS did not collect data on past-history of an OGTT (OGTT was performed in the SLHAS study itself). Furthermore, we believe it is unlikely that large numbers of people will have recent OGTT results readily available. Similarly, a history of a cholesterol test in the past 12 months was also not available in the SLHAS dataset and a cholesterol test was performed in the SLHAS study itself. A person who had a cholesterol test in the previous year would not require another cholesterol test, however our dataset limited us from modeling this. Furthermore, as the actual cholesterol level is ideally needed for CVD risk calculation, we modeled cholesterol testing for all people who did not report hypercholesterolemia and were not taking statins, even if some may have done cholesterol tests in the past 12 months. In reality, it is possible that only a small proportion of people would have recent cholesterol test results readily available.

Examples of people who would not qualify for screening in the model:

A 45-year-old with hypertension and taking a statin, but has no reported diagnosis of diabetes, is not on oral hypoglycemics or insulins, but had a blood glucose test in the past year does not qualify for screening.

A 50-year-old who reports having diabetes and is on a statin, but has no diagnosis of hypertension, and is not on an antihypertensive, but had a blood pressure measured in the past year does not qualify for screening.

Using binomial probability to calculate probability of being screened

We used binomial probabilities to calculate the probability that a participant would get the initial screen based on the number of OP visits reported over a 28-day period.

The formula used was

$$P_{assesed} = 1 - \left(\left(1 - P_{a\_pub}\right)^{n_{pub}} \times \left(1 - P_{a\_priv}\right)^{n_{priv}}\right)$$

Where P_{a_pub} is the probability of being assessed in the public sector

P_{a_pri} is the probability of being assessed in the private sector

n_{pub} is the number of visits to the public sector

n_{priv} is the number of visits to the private sector

For example, in the case where P_{a_pub} = 0.6 and P_{a_pri} = 0.55, a participant who had one public sector visit was given a probability of 0.6 of being screened, whilst a person with two public sector visits was assigned a probability of $1 - ((1 - 0.6)^2 \times (1 - 0.55)^0) = 0.84$.

Calculations for a yearly program

The SLHAS dataset used three separate recall periods for outpatient visits: Of the people aged 40 years and over with no CVD, the number of OP visits was asked from 812 people for a 7-day recall period, 780 people for a 14-day recall period and 2,380 people for a 28 day-recall period. There is no obvious way identify which proportion of people had at least one public OP visit or one private OP visit during the previous year, and of those who had either a public or private OP visit, how many visits were made to each. Presumably a proportion of people who reported zero visits in their respective recall period would have visited at least once over a 12-month period.

Given the lack of any other survey or administrative data available to i) estimate the proportion of people with at least one public or private OP visit in the past 12 months, and ii) the number of visits per person to each sector, we attempted to model this with our dataset.

We created a dataset of the proportion of people aged 40 years and over with zero public OP visits and zero private OP visits for each gender, sector (urban, rural, estate), socioeconomic quintile and recall period in days.

We then ran a regression:

$$prop_h = ß_1.\ln(day) + ß_2.sector + ß_3.sex + ß_4.sesquintile$$

Where *prop* is the proportion of the group that had no healthcare visits

h is the public or private health sector

day is the recall period in days

sector is urban, rural or estate/rural

sesquintile is the socioeconomic quintile.

Variables with coefficients with a p-value greater than 0.01 were dropped from the full model. The model confirmed an association of the proportion with no visits to the public sector with the number of days in the recall period and socioeconomic quintile (Supplementary Table 1). For the private sector, there was an association between the number of days in the recall period, sector and socioeconomic quintile (Supplementary Table 2).

Supplementary Table 1. Linear regression estimates of the proportion of people with no public outpatient visits with recall time period and sociodemographic factors

	Full model			Final model		
	**Coefficient**	**95% CI**	**p**	**Coefficient**	**95% CI**	**p**
Days (log)	-0.10	(-0.13, -0.08)	0.000	-0.10	(-0.13, -0.08)	0.000
Sector						
Rural	-0.02	(-0.06, 0.01)	0.196	*	*	*
Rural / Estate	-0.01	(-0.05, 0.02)	0.535	*	*	*
Female	-0.02	(-0.04, 0.01)	0.262	*	*	*
SES quintile						
2	0.00	(-0.04, 0.04)	0.997	0.00	(-0.04, 0.04)	0.997
3	0.02	(-0.02, 0.07)	0.268	0.02	(-0.02, 0.07)	0.267
4	0.05	(0.01, 0.10)	0.021	0.05	(0.01, 0.10)	0.021
5 (richest)	0.08	(0.04, 0.13)	0.000	0.08	(0.04, 0.13)	0.000
Constant	1.14	(1.06, 1.22)	0.000	1.12	(1.05, 1.20)	0.000
n	90			90		
Adj R^2	0.476			0.476		

Supplementary Table 2. Linear regression estimates of the proportion of people with no private outpatient visits with recall time period and sociodemographic factors

	Full model			Final model		
	**Coefficient**	**95% CI**	**p**	**Coefficient**	**95% CI**	**p**
Days (log)	-0.08	(-0.09, -0.06)	0.000	-0.08	(-0.09, -0.06)	0.000
Sector						
Rural	-0.01	(-0.03, 0.02)	0.641	-0.01	(-0.03, 0.02)	0.648
Rural / Estate	0.03	(0.01, 0.06)	0.017	0.03	(0.01, 0.06)	0.019
Female	-0.02	(-0.04, 0.00)	0.034	*	*	*
SES quintile						
2	-0.01	(-0.04, 0.03)	0.651	-0.01	(-0.04, 0.03)	0.658
3	-0.01	(-0.04, 0.03)	0.728	-0.01	(-0.04, 0.03)	0.734
4	-0.02	(-0.05, 0.01)	0.184	-0.02	(-0.06, 0.01)	0.193
5	-0.04	(-0.07, -0.01)	0.018	-0.04	(-0.07, -0.01)	0.021
Constant	1.15	(1.10, 1.21)	0.000	1.14	(1.09, 1.20)	0.000
n	90			90		
Adj R^2	0.479			0.456		

From the proportion of people who were estimated to have no visits during the previous year, we estimated the proportions of people in each sex, socioeconomic quintile and sector group who had one or more visits. We calculated the total number of visits for each group over a year based on the mean visits per person in each group for the recall period, scaled to one year, and divided it by the number of people with one or more visit to estimate the mean number of visits for each group. We then applied a Poisson distribution bounded by one, using the method outlined by Cohen (1960) using Molina's tables, to estimate the distribution of visits for each group given the calculated mean. The dataset was expanded so that one record was representative of one person, and the distribution of visits was randomly applied within each group.

Whilst recognizing that visits to a sector may cluster in individuals (for example, an individual may be more likely to have mostly private sector visits with minimal or no public sector visits), it is difficult to model how much overlap of public and private visits there would be over a course of a year. However, as visits were distributed within sector-SES or SES groups, with the chance of screening very similar in both sectors, and binomial probabilities resulting in a person with multiple visits having a high probability of being screened, the impact of the method of random distribution of visits used in this analysis is likely to be minimal.

The estimates generated are likely to be conservative, given that visits may cluster in time in individuals during a short recall period. Indeed, analysis of a weighted subsample of 131 people with an undiagnosed chronic condition, who were asked when they last had an OP visit, found that 66% of people had a public OP visit in the past year and 86% had a private OP visit (separate results, not shown), which is higher than the estimates generated from our model.

The remaining analysis for assessment, follow-up and diagnosis followed the same principles as the analysis using a 28-day timeframe.

References

Cohen AC. 1960. Estimating the parameter in a conditional Poisson distribution. *Biometrics*, **16**: 203-211.

Supplement 2 Wealth index

Supplementary Table 3. Assets used to calculate wealth index

Category	Details of assets included	
Durable assets	TV	
	VCD/DVD player	
	Sewing machine	
	Washing machine	
	Electric fan	
	Domestic phone	
	Mobile phone	
	Computer	
	Camera/Video camera	
	Bicycle	
	Motorcycle/Scooter	
	Three-wheeler	
	Motor car/Van	
	Bus, Lorry/Tipper	
Housing quality	Bedrooms in household	
	Main material used for floor	Cement, terrazzo or tile, concrete, mud, wood, sand, other
	Main material used for walls	Brick, cabok, cement block, pressed soil block, mud, wood or sheets, cadjan, palyrah or straw, other
Water and sanitization facilities	Main source of drinking water	Protected well within premises, protected well outside premises, unprotected well, river, natural spring, reservoir, tank, tap inside home, tap within premises, tap outside premises, project in village, tube well, bowser, rain water,
	Toilet facility	Exclusive to household, shared with another household, public convenience, no toilet facility
	Garbage disposal	Collected by garbage truck, burned, dumped within premises, processed for fertilizer, dumped or thrown away outside premises, other
Food storage and cooking facilities	Cooking place	In the house, in a separate building, outdoors, other
	Cooking fuel	Electricity, LP gas, natural gas, biogas, kerosene, coal or lignite, charcoal, wood, straw, shrubs or grass, agricultural crops, animal dung, no food cooked in house, other
	Fridge	
Other items	Household tenure type	Constructed/purchased & owned by an occupant, inherited & owned by an occupant, freely received/received as a gift & owned by occupant, compensated, rent free (employer/other), relief payment (employer/other), rent - government owned, rent - privately owned, lease - government owned, lease -privately owned, encroached, other
	Principal type of lighting	Kerosene, electricity, solar energy, battery or generator, gas, other
	Internet	

Supplement 3 Data tables

Supplementary Table 4. New diagnoses by program and chronic condition

Chronic condition	Total undiagnosed No. ('000s) (95% CI)	Newly diagnosed in 28-day program		Newly diagnosed in 1-year program	
		No. ('000s) (95% CI)	as % of total undiagnosed (95% CI)	No. ('000s) (95% CI)	as % of total undiagnosed (95% CI)
Any chronic condition	2,332 (2,148, 2,515)	192 (167, 217)	8.2 (6.8, 9.6)	628 (584, 671)	26.9 (26.5, 27.4)
Hypertension	1,413 (1,266, 1,559)	112 (92, 133)	8.0 (6.1, 9.8)	370 (335, 404)	26.2 (25.6, 26.8)
Hypercholesterolemia or high CVD risk	708 (599, 817)	62 (48, 75)	8.7 (6.2, 11.3)	195 (170, 221)	27.6 (27.2, 28.1)
Diabetes	770 (663, 877)	57 (45, 68)	7.4 (5.2, 9.6)	204 (177, 230)	26.5 (25.7, 27.2)

Supplementary Table 5. Sensitivity analysis: Number of people assessed, followed-up, and diagnosed by probability of assessment and follow-up, 28-day and 1-year program

Scenario	Probability of assessment (public %, private %)	Probability of follow-up (public %, private %)	Assessed No. ('000s) (95% CI)	Followed-up No. ('000s) (95% CI)	Males followed-up		Newly diagnosed cases	
					No. ('000s) (95% CI)	As % of followed-up group (95% CI)	No. ('000s) (95% CI)	As % of undiagnosed cases‡ (95% CI)
28-day program	60, 55	80, 80	1,412 (1,292, 1,532)	889 (820, 957)	373 (329, 417)	42 (37, 47)	256 (207, 304)	11.0 (9.1, 12.8)
28-day program *	60, 55	60, 60	1,412 (1,292, 1,532)	666 (615, 718)	280 (247, 312)	42 (37, 47)	192 (156, 228)	8.2 (6.8, 9.6)
28-day program	60, 55	40, 40	1,412 (1,292, 1,532)	444 (410, 479)	186 (165, 208)	42 (37, 47)	128 (104, 152)	5.5 (4.6, 6.4)
1-year program	30, 28	80, 80	3,683 (3,587, 3,778)	2,557 (2,479, 2,634)	1,147 (1,098, 1,195)	45 (43, 47)	837 (778, 895)	35.9 (35.3, 36.5)
1-year program *	30, 28	60, 60	3,683 (3,587, 3,778)	1,918 (1,859, 1,976)	860 (824, 896)	45 (43, 47)	628 (584, 671)	26.9 (26.5, 27.4)
1-year program	30, 28	40, 40	3,683 (3,587, 3,778)	1,278 (1,240, 1,317)	573 (549, 598)	45 (43, 47)	418 (389, 448)	17.9 (17.6, 18.2)
1-year program	60, 55	80, 80	4,279 (4,170, 4,387)	2,972 (2,883, 3,060)	1,391 (1,333, 1,449)	47 (45, 49)	976 (909, 1,043)	41.9 (41.2, 42.5)
1-year program	60, 55	60, 60	4,279 (4,170, 4,387)	2,229 (2,162, 2,295)	1,043 (1,000, 1,087)	47 (45, 49)	732 (681, 783)	31.4 (30.9, 31.9)
1-year program	60, 55	40, 40	4,279 (4,170, 4,387)	1,486 (1,441, 1,530)	696 (667, 725)	47 (45, 49)	488 (454, 522)	20.9 (20.6, 21.2)

Notes:
* Base-case 28-day program and 1-year program
‡ Reported as a percentage of all people with an undiagnosed chronic condition, regardless of whether they reported an OP visit (2,331,757 people).

Supplementary Table 6. Sensitivity Analysis: Costs of screening program by probability of assessment and follow-up, 28-day and 1-year program

Scenario	Probability of assessment (public %, private %)	Probability of follow-up (public %, private %)	Cost per person screened (USD) (95% CI)		Cost per person diagnosed (USD) (95% CI)		Total cost ('000 USD) (95% CI)		Cost as % of annual expenditure (%) (95% CI)	
			Public	Private	Public	Private	Public	Private	Public	Private
28-day program	60, 55	80, 80	1.28 (1.14, 1.43)	3.15 (2.76, 3.55)	6.71 (5.94, 7.47)	19.04 (16.63, 21.44)	1,100 (974, 1,226)	1,751 (1,530, 1,972)	0.09 (0.08, 0.10)	0.14 (0.12, 0.16)
28-day program *	60, 55	60, 60	1.01 (0.90, 1.13)	2.64 (2.32, 2.97)	7.05 (6.24, 7.85)	21.27 (18.67, 23.88)	867 (768, 966)	1,467 (1,288, 1,647)	0.07 (0.06, 0.08)	0.12 (0.10, 0.13)
28-day program	60, 55	40, 40	0.74 (0.65, 0.82)	2.13 (1.88, 2.38)	7.73 (6.84, 8.61)	25.75 (22.73, 28.76)	634 (561, 706)	1,184 (1,045, 1,323)	0.05 (0.05, 0.06)	0.10 (0.09, 0.11)
1-year program	30, 28	80, 80	1.47 (1.43, 1.52)	3.41 (3.30, 3.52)	6.47 (6.27, 6.67)	15.08 (14.59, 15.57)	3,231 (3,131, 3,331)	5,087 (4,923, 5,251)	0.27 (0.26, 0.28)	0.41 (0.40, 0.43)
1-year program *	30, 28	60, 60	1.17 (1.13, 1.20)	2.87 (2.78, 2.96)	6.82 (6.61, 7.03)	16.92 (16.37, 17.47)	2,555 (2,476, 2,634)	4,281 (4,143, 4,419)	0.21 (0.21, 0.22)	0.35 (0.34, 0.36)
1-year program	30, 28	40, 40	0.86 (0.83, 0.88)	2.33 (2.25, 2.40)	7.53 (7.29, 7.76)	20.60 (19.94, 21.26)	1,879 (1,821, 1,938)	3,475 (3,363, 3,587)	0.16 (0.15, 0.16)	0.28 (0.27, 0.29)
1-year program	60, 55	80, 80	1.47 (1.43, 1.52)	3.41 (3.31, 3.52)	6.44 (6.25, 6.63)	15.03 (14.56, 15.50)	3,745 (3,632, 3,857)	5,930 (5,744, 6,116)	0.31 (0.30, 0.32)	0.48 (0.47, 0.50)
1-year program	60, 55	60, 60	1.17 (1.13, 1.20)	2.87 (2.78, 2.96)	6.79 (6.59, 6.99)	16.86 (16.33, 17.39)	2,961 (2,872, 3,050)	4,989 (4,833, 5,145)	0.25 (0.24, 0.26)	0.41 (0.39, 0.42)
1-year program	60, 55	40, 40	0.86 (0.83, 0.88)	2.33 (2.26, 2.40)	7.49 (7.26, 7.72)	20.52 (19.87, 21.16)	2,177 (2,112, 2,243)	4,048 (3,921, 4,175)	0.18 (0.18, 0.19)	0.33 (0.32, 0.34)

Notes:
Annual Health Expenditure in 2019 estimated at USD 1,195 million in the public sector and USD 1,228 million in the private sector converted to December 2021 US dollars (US$1=LKR 201.40).
Source: Amarasinghe S, Dalpatadu K, Rannan-Eliya R. Sri Lanka Health Accounts: National Health Expenditure 1990-2019. Colombo: Institute for Health Policy; 2021.

Supplementary Table 7. Distributions of screening eligible, undiagnosed and newly diagnosed individuals by socioeconomic status with 28-day screening program (number of participants)

SES quintile	Eligible for screening† No. ('000s) (95% CI)	Eligible for screening with ≥ 1 chronic condition	Undiagnosed		Newly diagnosed		
			Before screening	After screening	Public	Private	Both
		No. ('000s) (95% CI)	No. ('000s) (95% CI)	No. ('000s) (95% CI)	No. ('000s) (95% CI)	No. ('000s) (95% CI)	No. ('000s) (95% CI)
1 (poorest)	1,536 (1,424, 1,647)	901 (794, 1,009)	499 (409, 589)	449 (375, 523)	39 (21, 57)	11 (1, 22)	50 (29, 71)
2	1,533 (1,423, 1,642)	901 (795, 1,007)	446 (358, 533)	413 (334, 492)	25 (14, 36)	8 (0, 15)	33 (20, 46)
3	1,528 (1,428, 1,628)	961 (858, 1,065)	478 (392, 564)	443 (367, 519)	25 (11, 39)	11 (3, 18)	36 (20, 52)
4	1,531 (1,427, 1,636)	1,002 (892, 1,111)	458 (370, 545)	414 (338, 491)	25 (11, 38)	18 (8, 28)	43 (27, 60)
5 (richest)	1,532 (1,417, 1,647)	1,074 (958, 1,191)	451 (363, 539)	421 (341, 500)	10 (1, 18)	21 (9, 33)	30 (16, 45)
Total	7,660 (7,418, 7,902)	4,840 (4,596, 5,083)	2,332 (2,136, 2,528)	2,140 (1,968, 2,311)	123 (93, 153)	69 (47, 91)	192 (156, 228)
Concentration index (95% CI)							
	Eligible with ≥ 1 chronic condition‡	Not applicable	-0.106 (-0.164, -0.049) p<0.001	-0.089 (-0.143, -0.035) p=0.001	-0.027 (-0.038, -0.016) p<0.001	0.010 (0.002, 0.018) p=0.013	-0.017 (-0.030, -0.004) p=0.010
	Eligible*	0.100 (0.055, 0.145) p<0.001	-0.019 (-0.062, 0.023) p=0.375	-0.012 (-0.052, 0.027) p=0.541	-0.014 (-0.021, -0.008) p<0.001	0.008 (0.003, 0.013) p=0.003	-0.007 (-0.015, 0.002) p=0.111

Notes: †People aged 40 years and over without pre-existing CVD. ‡Concentration index calculated on all people aged 40 years and over without pre-existing CVD, and with at least one chronic condition. * Concentration index calculated on all people aged 40 years and over without pre-existing CVD.
SES = socioeconomic status
